# Mycorrhizosphere Bacteria, *Rahnella* sp. HPDA25, Promotes the Growth of *Armillaria gallica* and Its Parasitic Host *Gastrodia elata*

**DOI:** 10.3389/fmicb.2022.842893

**Published:** 2022-03-17

**Authors:** Tianrui Liu, Zhongyi Hua, Pengjie Han, Yuyang Zhao, Junhui Zhou, Yan Jin, Xiaolin Li, Luqi Huang, Yuan Yuan

**Affiliations:** ^1^State Key Laboratory of Dao-di Herbs, National Resource Center for Chinese Materia Medica, China Academy of Chinese Medical Sciences, Beijing, China; ^2^School of Pharmaceutical Sciences, Peking University, Beijing, China

**Keywords:** plant growth-promoting bacteria, orchid mycorrhizal symbiosis, cultivation methods, *Rahnella*, *Armillaria*

## Abstract

*Gastrodia elata* is an entirely heterotrophic plant, the growth of which is completely reliant on *Armillaria gallica*, an orchid mycorrhizal fungus. To avoid damaging ecosystems, *G. elata* cultivation is shifting from woodland to farmland. However, whether the microbial community structure remains stable during this conversation is unknown. Here, we cultivated *G. elata* in woodland or farmland and found that woodland-cultivated *G. elata* produced a greater yield and larger tuber size. The relative abundance of *Rahnella* was 22.84- and 122.25-fold higher in woodland- and farmland-cultivated soil samples, respectively, than that in uncultivated soil samples. To investigate how *Rahnella* impacts the growth of *G. elata* and establishes symbiosis with *Armillaria gallica*, three *Rahnella* spp. strains (HPDA25, SBD3, and SBD11) were isolated from mycorrhizosphere soil samples. It was found that these strains, especially HPDA25, promoted the growth of *A. gallica*. Ultra-performance liquid chromatography coupled to a triple quadrupole mass spectrometry analysis detected the indole-3-acetic acid with 16.24 ng/ml in HPDA25 fermentation solution. Co-culturing with the strain HPDA25 or exogenous indole-3-acetic acid increased the branching and fresh weight of rhizomorphs and the growth rate and extracellular laccase activity of *A. gallica*, compared with *A. gallica* cultured alone. The results of RNA-seq and quantitative real-time polymerase chain reaction analysis showed that co-culturing *A. gallica* with HPDA25 increased the expression level of the genes including hydrophobin, SUR7/PalI family, and pectin methylesterase, whereas decreased the expression levels of glycolysis-related genes. Furthermore, co-culturing with the strain HPDA25, *A. gallica* promotes the growth of *G. elata* and enhances the tuber size of *G. elata*. These results provide new insights into an orchid mycorrhizal symbiosis and the cultivation of *G. elata*.

## Introduction

Plants establish associations with mutualistic fungi and bacteria to exchange key nutrients and thrive (Wang et al., 2021). Orchidaceae, one of the most diverse and widely distributed plant families, is partially or fully dependent on mycorrhizal fungi during the life cycle (Lu et al., 2019). The popular herb *Gastrodia elata*, a member of the orchid family, is widely distributed in Asian countries such as South Korea, Japan, and China (Chen et al., 2019). As a completely heterotrophic plant, *G. elata* spends more than 80% of its life underground as a tuber, associating with the mycorrhizal fungus *Armillaria* to supply the required nutrition (Yuan et al., 2018). *Armillaria* is well known as a contributor to carbon cycling *via* woody tissue breakdown (Alveshere et al., 2021) and is used in *G. elata* cultivation in China.

Abundant biodiversity in the plant environment leads to a productive ecosystem, improving yield and agricultural sustainability (Reiss and Drinkwater, 2018). For example, core microbial communities increased the drought tolerance of *Adenium obesum*, *Aloe dhufarensis*, and *Cleome austroarabica* (Khan et al., 2020). The isolation and culture of potentially beneficial microorganisms could increase *Panax notoginseng* yields in a continuous cropping system (Li et al., 2020). *G. elata* interacts with a diverse spectrum of bacteria during its growth before the arrival of *Armillaria* (Yuan et al., 2018). We hypothesize that bacteria also play an important role in the establishment of a symbiotic relationship between *Armillaria gallica* and *G. elata*. However, the dynamics of bacterial communities during the growth of *G. elata* under different environments is unknown.

Plant growth-promoting bacteria (PGPB) have been extensively described in the context of sustainable agricultural systems, which directly or indirectly promote plant growth (Hassan, 2017). As important beneficial microorganisms, PGPB play vital roles in improving plant growth and crop production (Ha-Tran et al., 2021). The mycorrhizosphere’s bacterial and fungal communities are extremely varied and complicated (Wagg et al., 2019), influencing plant development and symbiosis (Herrera et al., 2020). Co-inoculation with PGPB and arbuscular mycorrhizal (AM) fungi increased nutrient uptake in plants, leading to high crop yields (Emmanuel and Babalola, 2020), supporting the idea that crop yields may be positively correlated with soil microbial richness (Kieck et al., 2016). However, how mycorrhizosphere bacteria affect the growth of *G. elata* and *A. gallica* is poorly understood.

Apart from their roles in the plant–fungus symbiosis, root-associated bacteria also contribute to plant fitness, variety, and coexistence *via* bi- or tripartite interactions between plant hosts and mycorrhizal fungi (Kaur and Sharma, 2021). Temperature, pH, soil type, vegetation, and landscapes could influence the microbial populations in the soil (Finkel et al., 2017), and the plant diversity also increases the diversity of soil microorganisms (Stefan et al., 2021). Till now, a final conclusion has not yet been reached on the function of the bacterial communities during the development phases of *G. elata* and *A. gallica*.

Here, we compared the mycorrhizosphere bacterial communities, agronomic traits, and yield of *G. elata* cultivated in woodland or farmland. We aimed to understand the dynamics of the mycorrhizosphere bacteria associated with *G. elata* and *A. gallica* under different cultivation environments and the function of mycorrhizosphere bacteria in the growth of *G. elata* and *A. gallica*. We also tried to isolate growth-promoting mycorrhizosphere bacteria and to investigate the mechanisms of orchid mycorrhizal (OM) microbiome associations.

## Materials and Methods

### Cultivation, Harvest, and Agronomic Traits Analysis of *G. elata*

**FIGURE 1:**
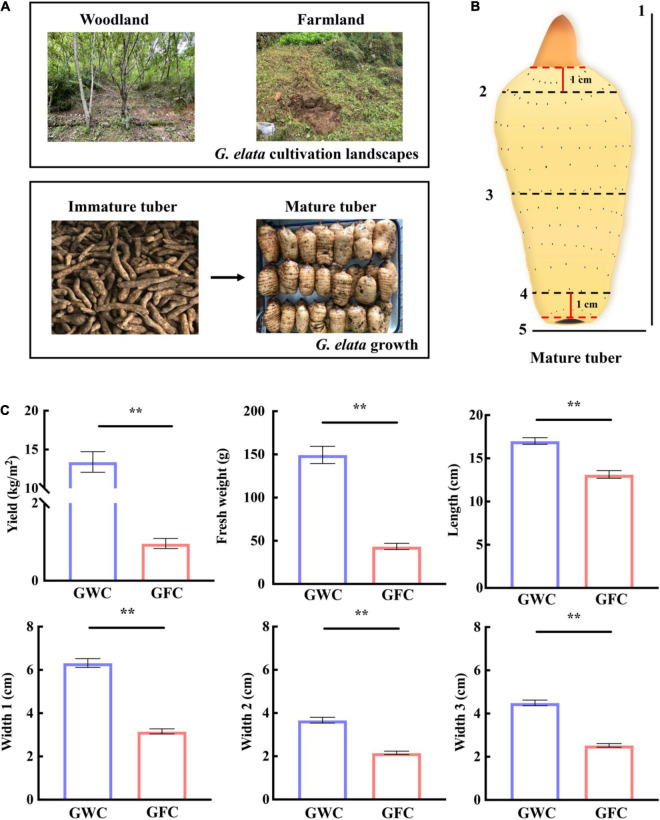
*G. elata* cultivation, growth, and evaluation of mature tuber agronomic traits. **(A)** Cultivation of *G. elata* in woodland or farmland. **(B)** Agronomic traits of *G. elata* mature tubers. 1, length; 2, width at 1 cm from top bud (Width 2); 3, half of tuber width (Width 1); 4, width at 1 cm from tuber bottom (Width 3); 5, weight. **(C)** Yield, tuber weight, and agronomic trait evaluation of *G. elata*. GWC, *G. elata* cultivated in woodland; GFC, *G. elata* cultivated in farmland. Data are shown as means ± SE. **P < 0.01.

*Gastrodia elata* was planted and harvested in woodland or farmland landscapes (33°309″N, 108°828″E) in Ningshan (Shaanxi Province, China) in 2019. A total of 16 plant holes, 30 cm (length) × 30 cm (width) × 70 cm (depth), were dug in each landscape. In March, five wood sticks (diameter = 5–12 cm, length = 30 cm) fully infected with *A. gallica* were placed in parallel at the bottom of each plant hole, in which 0.2 kg/m^2^ of *G. elata* tubers were planted. The cultivation and production processes are shown in Figure 1A. In November, mycorrhizosphere soil samples, including the surface soil samples of farmland (FC)- and woodland (WC)-cultivated *G. elata* mature tuber and *A. gallica* rhizomorphs, were collected. Uncultivated soil samples from farmland (UNFC) and woodland (UNWC) were collected as control. Meanwhile, the yield of *G. elata* was evaluated, and agronomic traits such as weight, length, and width in each mature tuber (Figure 1B) were measured. For agronomic characteristics analysis, a total of 121 mature tubers were measured in woodland, and 119 mature tubers were measured in farmland.

### Diversity of Bacterial Community

The total genomic DNA was extracted from mycorrhizosphere soil samples using the CTAB method. Each landscape was sampled for a total of 16 soil samples. 16S rRNA genes were amplified using the specific primers 515F (5′-GTGCCAGCMGCCGCGGTAA-3′) and 806R (5′-GGACTACHVGGGTWTCTAAT-3′) (Caporaso et al., 2011). All polymerase chain reaction (PCR) reactions were carried out using Phusion^®^ High-Fidelity PCR Master Mix (New England Biolabs, Ipswich, MA, United States). PCR products were purified with the Qiagen Gel Extraction Kit (Qiagen, Hilden, Germany). Sequencing libraries were generated using the TruSeq^®^ DNA PCR-Free Sample Preparation Kit (Illumina, San Diego, CA, United States) following the manufacturer’s recommendations. Library quality was assessed using a Qubit^®^ 2.0 Fluorometer (Thermo Fisher Scientific, Waltham, MA, United States) and Agilent Bioanalyzer 2100 system (Agilent Technologies, Santa Clara, CA, United States). The library was sequenced using the Illumina HiSeq 2500 platform, and 250-bp paired-end reads were generated.

FASTQ files from each sample were processed using Cutadapt version V1.9.1 software^1^ (Langille et al., 2013) for preliminary quality control of raw data. Briefly, paired-end reads were joined, depleted of barcodes, and trimmed, and chimeras were removed. The clean sequencing reads were clustered into operational taxonomic units (OTUs) with 97% identity using UPARSE version 7.0.1001 software^2^ (Haas et al., 2011). According to the algorithm principle, sequences with the highest frequencies were selected as the representative sequences of OTUs.

QIIME2 software (Hall and Beiko, 2018) and the SSU rRNA database (Wang et al., 2007) of SILVA 132^3^ (Edgar, 2013) were used to perform species annotation analysis of the OTU sequences (set threshold 0.8–1) to obtain taxonomic information and statistics of the community composition of each sample at each classification level (kingdom, phylum, class, order, family, genus, and species). MUSCLE version 3.8.31 software (Quast et al., 2013)^4^ was used to perform rapid multiple sequence alignment to obtain the phylogenetic relationships of all OTU sequences.

Finally, data were normalized to that of the sample with the least amount of data. The subsequent diversity of bacterial community analyses and Spearman correlation analysis between bacterial community and agronomic traits of *G. elata* tuber were performed in NovoMagic v3.0^5^.

### Isolation and Identification of Mycorrhizosphere Bacteria

The soil on the tubers was rinsed off with Milli-Q water, and the tubers were then ultrasonically cleaned three times (120 W, 40 kHz, 5 min). The fresh tubers were subsequently rinsed three times with sterile water, cut into small pieces, and homogenized with 2-ml sterile water. A final 10^6^-fold diluted suspension was prepared using sterile water, and 100 μl of the tissue suspension was plated onto various media, including potato dextrose agar (PDA), Luria-Bertani medium (LB), tryptone soya agar, and nutrient agar. Plates were incubated at 30°C for 2–5 days or until bacterial growth was observed (Luo et al., 2019). All experiments were performed in triplicate. LB medium was used for subsequent purification, growth, and maintenance of bacterial strains. All isolated strains were purified and stored in 50% glycerol at −80°C.

Amplification of the 16S rRNA gene was performed using the primers 27F (5′-CAGAGTTTGATCCTGGCT-3′) and 1492R (5′-AGGAGGTGATCCAGCCGCA-3′) (Ying et al., 2012). The PCR mixture (50 μl) contained 2-μl DNA template, 25-μl 2 × M5 HiPer Taq Mix (Mei5bio, Beijing, China), 19-μl ddH_2_O, and 2 μl of each primer (10 μM). Amplification of the 16S rRNA genes was performed using the following thermal cycling steps: initial denaturation at 94°C for 10 min, followed by 35 cycles of denaturation at 94°C for 30 s, annealing at 55°C for 30 s, extension at 72°C for 1 min, and a final extension at 72°C for 10 min. The amplified products were purified and sequenced by BGI Genomics (Beijing, China). The obtained sequences were identified using EzBioCloud^6^ and the National Center for Biotechnology Information BLAST^7^. Multiple sequence alignments and genetic distance calculations were performed using the CLUSTAL X program (Larkin et al., 2007), and a neighbor-joining phylogenetic dendrogram was generated using MEGA X software (Kumar et al., 2018). The 16S rRNA gene sequences determined in this study were deposited in GenBank under accession numbers OL700214–OL700216.

### Co-cultivation Assay of Mycorrhizosphere Bacterial Strains and *A. gallica*

*Armillaria gallica* strain for the co-cultivation assay was previously isolated from *G. elata* tubers and preserved in the laboratory. *A. gallica* was incubated at 25°C in the dark for 14 days. The stored bacterial strains were incubated in a solid LB medium at 28°C for 12 h. Fresh rhizomorphs were used for the co-culture experiments. The isolated mycorrhizosphere bacterial strains were cultured in a liquid LB medium at 28°C to an optical density at a wavelength of 600 nm of approximately 0.5 (1.47 × 10^10^CFU/ml for HPDA25, 1.87 × 10^10^ CFU/ml for SBD3, and 1.73 × 10^10^ CFU/ml for SBD11). After that, 100 μl of bacterial liquid was co-cultured with *A. gallica* on a PDA medium in the dark for 7 days at 25°C. As controls, *A. gallica* was cultured without bacterial liquid. After separating *A. gallica* from the medium, the fresh rhizomorph’s weight, growth rate, branching, and extracellular laccase activity of *A. gallica* were assessed. Six biological replicates were performed for each condition. Separating *A. gallica* rhizomorphs was used as extracellular laccase activity, indole-3-acetic acid (IAA) content determination, transcriptome sequencing, and quantitative polymerase chain reaction (qPCR).

### Extracellular Laccase Activity Analysis

In separating *A. gallica* from the medium, the medium was weighted (approximately 4 g) and centrifuged at 13,000 rpm for 15 min. The supernatant (1 ml) was then diluted with Milli-Q water to a final volume of 10 ml for extracellular laccase activity determination. The extracellular laccase activity was measured using the 2,2-azino-bis-3-ethylbenzothiazoline-6-sulfonic acid (ABTS) method (Quijada-Morin et al., 2018). The reaction system contained 100 μl of 0.2 mol/L disodium hydrogen phosphate-citric acid buffer (pH = 4.0), 50-μl ABTS, and 50-μl dilute enzyme solution. The absorbance of ABTS and enzyme solution mixture was measured 10 times using a microplate reader (Thermo Fisher Scientific) at 420 nm. One unit of laccase activity was defined as the amount of laccase required to catalyze the oxidation of 1-μmol ABTS per minute in the reaction system. Six biological replicates were performed for each condition.

### Indole-3-Acetic Acid Content Determination

HPDA25 was cultured in 20 ml of LB medium at 28°C and 200 rpm until the optical density at a wavelength of 600 nm of approximately 0.5 (1.47 × 10^10^ CFU/ml). After that, the fermentation solution was centrifuged at 12,000 rpm for 15 min, and the supernatant was removed under nitrogen gas. Subsequently, 70% methanol was added to obtain a final volume of 10 ml, and the mixture was passed through a 0.22-μm filter. Approximately 0.1 g separating *A. gallica* rhizomorphs was used as IAA content determination.

Indole-3-acetic acid (CAS: 87-51-4) standards were purchased from MedChemExpress (Monmouth Junction, NJ, United States), and hormone levels were determined using ultra-performance liquid chromatography coupled to a triple quadrupole mass spectrometry. The mobile phase consisted of 0.1% formic acid water (B) and acetonitrile (A) (v/v) with the following gradient: 10% A (0–0.3 min), 10–60% A (0.3–3 min), 60–95% A (3–6 min), 10% A (6–6.2 min), and 10% A (6.2–7 min). The equilibration time and the flow rate were set at 7 min and 0.5 ml/min, respectively. The ACQUITY UPLC BEH C_18_ column (2.1 mm × 100 mm, 1.7 μm; Waters) was used with a temperature of 40°C, sample plate temperature of 4°C, a sample volume of 1 μl, and analysis time of 7 min. Electrospray ionization was used with 176.000/130.100 (m/z) (ESI+) and 174.000/130.100 (m/z) (ESI−) for IAA (Jiang et al., 2017). The scanning mode captured positive and negative ion data using multi-reaction detection. The pressure of the curtain gas was 30 psi, the ionization voltage was −4,500 V/+5,500 V, the spray gas pressure was 50 psi, the auxiliary heating gas pressure was 50 psi, the acquisition method was +-MRM, and the ion source temperature was 550°C.

### Growth of *A. gallica* in the Medium Containing Indole-3-Acetic Acid

Approximately 16.24 ng/ml IAA was added to the PDA medium, and the *A. gallica* rhizomorphs were cultured in a 25-ml IAA-containing PDA medium. Also, the control group was cultured in a 25-ml PDA medium supplemented with 70% methanol solution. *A. gallica* was incubated at 25°C in the dark for 7 days, and the fresh rhizomorph’s weight, growth rate, branching, and extracellular laccase activity of *A. gallica* were measured. Five biological replicates were performed for each condition.

### Transcriptome Sequencing and Quantitative Polymerase Chain Reaction of Co-cultured *A. gallica* With HPDA25

Fresh *A. gallica* rhizomorphs were isolated from the medium. RNA was extracted from three biological replicates of cultivated *A. gallica* rhizomorphs. The RNA integrity was assessed using the Bioanalyzer 2100 system (Agilent Technologies, CA, United States). Sequencing libraries were constructed using the Next Ultra RNA Library Prep Kit for Illumina (New England Biolabs). The 150-bp paired-end reads were then generated by Illumina HiSeq 4000 platform. Quality control of RNA-seq reads was performed using FastQC software (default parameters) (Wang et al., 2015; Ewels et al., 2016). Quality-filtered reads were aligned to the *A. gallica* genome GWHASIQ00000000 [National Genomics Data Center (NGDC), China]^8^ using STAR software (Dobin et al., 2013). Read counts were obtained with featureCounts v1.5.0-p3. Differential expression analysis was performed using the DESeq2 R package (1.20.0). The transcript levels with adjusted *P*-value ≤ 0.05 and a fold change (log2FC) ≥1 were designated as significant differentially expressed genes (DEGs; Fradj et al., 2020). Each DEG was annotated using the Gene Ontology (GO) and Kyoto Encyclopedia of Genes and Genomes, and the enrichment analysis was performed using the clusterProfiler R package (Wu et al., 2021).

We used quantitative real-time polymerase chain reaction (qRT-PCR) to validate the DEGs that were enriched by GO and Kyoto Encyclopedia of Genes and Genomes annotation. The primers of reference gene (18S) and DEG primers were designed using the Primer3Plus software^9^ and synthesized by BGI Genomics (Beijing, China) (Supplementary Table 1). *A. gallica* RNA was reverse transcribed to cDNA using the TransScript II First-Strand cDNA Synthesis SuperMix Kit (TransGen Biotech, Beijing, China) according to the manufacturer’s instructions. The cDNA was diluted to 200 μl with sterile water. Tip Green qPCR SuperMix Kit (TransGen Biotech, Beijing, China) was used to conduct RT-PCR on LightCycler 480 II (Roche Applied Science, Mannheim, Germany). Each RT-PCR mixture contained 2-μl cDNA, 1-μl primers, 5-μl Tip Green qPCR SuperMix, and 2-μl nuclease-free water. The qRT-PCR conditions were as follows: initial denaturation at 94°C for 30 s, followed by denaturation at 94°C for 5 s and annealing of primers at 60°C for 20 s, and extension at 72°C for 20 s. The samples were cooled to 60°C and then heated to 94°C by 40 cycles, and the melting curves were generated. Delta delta *C*t was used for statistical analysis. Three replicates were performed.

### HPDA25 Co-cultured With *A. gallica* and *G. elata*

Short-cut woods were sterilized at 121°C for 120 min. Tubers of *G. elata* were surface sterilized using the following three steps: washing in 75% ethanol for 30 s, in 2% NaOCl for 15 min, followed by rinsing 10 times in sterile distilled water. To verify the effectiveness of the surface sterilization, the distilled water from the final rinse was plated in LB medium and then incubated at 25°C for 5 days. Surface sterilization was considered effective when no microbial growth was observed in the medium. First, HPDA25 fermentation solution (300 μl) and 1% agar were mixed in 100-ml distilled water as the medium. The same volume (300 μl) of LB medium and 1% agar were used as control. Then, the sterilized wood that interacted with *A. gallica* rhizomorphs for 20 days and surface-sterilized tubers were added to this medium. Finally, another 100-μl HPDA25 fermentation solution was added to the medium surface and cultured at 25°C for 30 days (Khalil et al., 2021). In the control treatment, 100-μl LB medium was also applied to the medium surface. Six biological replicates were performed for each condition.

### Statistical Analysis

All statistical analysis was performed using IBM SPSS version 21.0 (IBM Corp., Armonk, NY, United States). Comparisons between control and treatment groups were analyzed by the Student’s *T*-test, and comparisons for more than two treatments were performed using one-way analysis of variance followed by Duncan’s *post hoc* test. Data are presented as means ± SEM. The analysis of similarities was performed using NovoMagic v3.0 (see text footnote 5). Graphs were constructed using GraphPad Prism 9 software (GraphPad Software, La Jolla, CA, United States), TBtools (Chen et al., 2020), and Hiplot^10^, a comprehensive web platform for scientific data visualization.

### Data Availability

The 16S sequence datasets are available at the NGDC Genome Sequence Archive BioProject number PRJCA005506^11^. The transcriptome dataset can be found at the NGDC Genome Sequence Archive BioProject number PRJCA005508^12^.

## Results

### Yield and Agronomic Traits of *G. elata*

*Gastrodia elata* was first cultivated in woodland and then transferred to farmland for protecting the ecosystem in recent years in China. Here, we tried to compare the effect of two cultivation methods on the growth of *G. elata* (Figure 1A). For a clear description of the mature tuber, three tuber widths were measured, including the width at half of the tuber (Width 1), the width at 1 cm from the top bud (Width 2), and the width at 1 cm from the tuber bottom (Width 3) (Figure 1B). When cultivated in woodland (GWC), the average fresh weight of one *G. elata* tuber was 149.51 g, and the yield can reach 13.40 kg/m^2^, which were 3.43-fold and 13.97-fold higher than those cultivated in farmland (GFC). The lengths and widths (Width 1–3) of woodland-cultivated tubers were also larger than those of farmland-cultivated tubers, and Width 1 of woodland-cultivated tubers were twofold larger than that of farmland-cultivated tubers (Figure 1C). These results showed that *G. elata* cultivated in woodland produced heavier, longer, and wider tubers than *G. elata* cultivated in farmland, leading to a higher yield.

### Diversity, Isolation, and Identification of Mycorrhizosphere Bacteria

Although the dynamics of fungal communities during *G. elata* growth have been reported (Chen et al., 2019), the bacterial communities are unknown. To further investigate whether the bacterial communities affect the tuber growth of *G. elata*, the diversity of mycorrhizosphere bacteria under two cultivation environments was investigated based on 16S rRNA gene sequencing. A total of 5,674 OTUs were used for diversity analysis. The sequenced samples were sufficient to reveal the true diversity because the Good’s coverage was higher than 0.97 in all soil samples (Supplementary Table 2). The result of non-metric multidimensional scaling and analysis of similarities showed a clear separation (stress = 0.098) and the differences of bacterial communities between WC/FC and UNWC/UNFC soil samples (Supplementary Figure 1 and Supplementary Table 3), suggesting *G. elata* could regulate the composition and abundance of mycorrhizosphere bacterial communities. The results also showed that the bacterial community diversity in uncultivated soil samples (UNWC, UNFC) was significantly higher than that in cultivated soil samples (WC, FC) (Supplementary Table 2).

**FIGURE 2:**
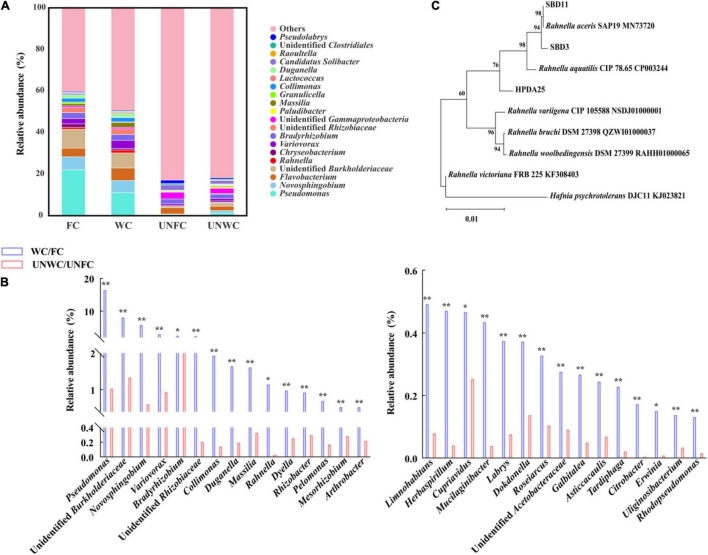
Mycorrhizosphere bacterial diversity. **(A)** Mycorrhizosphere bacteria whose relative abundance was in top 30 at genus levels. **(B)** Top 30 genera with a significantly increased relative abundance in WC/FC soil samples compared with that in UNWC/UNFC soil samples. **(C)** Phylogenetic tree (neighbor-joining) of *Rahnella* isolates. WC, *G. elata* cultivated mycorrhizosphere soil from woodland cultivating *G. elata*; FC, mycorrhizosphere soil from farmland cultivating *G. elata*; UNWC, mycorrhizosphere soil from woodland uncultivating *G. elata*; UNFC, *G. elata* cultivated mycorrhizosphere soil from farmland uncultivating *G. elata*. *P < 0.05; **P < 0.01.

The relative abundance of the 14 genera, including *Pseudomonas*, *Novosphingobium*, *Flavobacterium*, *Rahnella, Variovorax*, *Bradyrhizobium*, *Massilia*, *Collimonas*, *Duganella*, *Raoultella*, *Limnohabitans*, *Dyella*, *Rhizobacter*, and *Mucilaginibacter*, were increased in WC soil samples compared with that in UNWC soil samples and were also increased in FC soil samples compared with that in UNFC soil samples (Figures 2A,B and Supplementary Table 4). Furthermore, 11 genera among these 14 genera were associated with the enhancement of tuber growth in *G. elata* (Supplementary Figure 2). In particular, the relative abundance of genus *Rahnella* was 22.84-fold higher in WC soil samples than that in UNWC soil samples and 122.25-fold higher in FC soil samples than that in UNFC soil samples (Supplementary Table 4), suggesting that *Rahnella* could be involved in the growth promotion of *G. elata* tuber and that the relative abundance of *Rahnella* is affected by the cultivation environments.

### *Rahnella* spp. Co-cultured With *A. gallica* and *G. elata*

**FIGURE 3:**
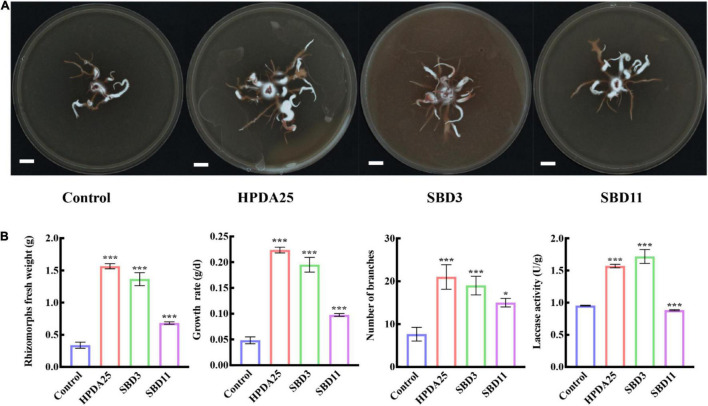
*Rahnella* spp. co-cultured with *A. gallica*. **(A)** *A. gallica* co-cultured with three *Rahnella* spp. **(B)** *Rahnella* spp. increased rhizomorph’s fresh weight, growth rate, branching, and extracellular laccase activity of *A. gallica*. Control, *A. gallica* cultured alone in medium. Data are shown as means ± SE. *P < 0.05; ***P < 0.001.

To verify the growth promotion on *G. elata* tuber of these *Rahnella* spp., three *Rahnella* spp. strains (HPDA25, SBD3, and SBD11) were isolated from mycorrhizosphere soils (Figure 2C) and identified using 16S rRNA sequences (Supplementary Table 5). We co-cultured these three *Rahnella* spp. strains (HPDA25, SBD3, and SBD11) with *A. gallica* and *G. elata*, respectively. The results showed that co-cultivation with HPDA25 or SBD3 increased the fresh weight of rhizomorphs (4.62- and 4.03-fold, respectively), growth rate (4.63- and 4.03-fold, respectively), rhizomorph branching (2.74- and 2.48-fold, respectively), and extracellular laccase activity (1.64- and 1.81-fold, respectively). SBD11 increased the fresh weight of rhizomorphs (2.02-fold), growth rate (2.02-fold), and rhizomorph branching (1.95-fold) and decreased extracellular laccase activity (0.93-fold) of *A. gallica* (Figures 3A,B). Among the three strains of *Rahnella* spp., HPDA25 was the predominant strain involved in the growth promotion of *A. gallica*.

**FIGURE 4:**
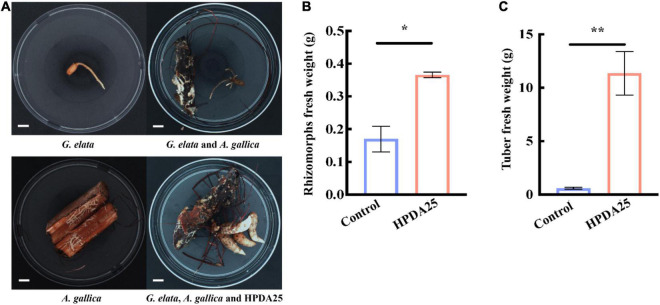
HPDA25 promoted both *A. gallica* and *G. elata* growth. **(A)** HPDA25, *A. gallica*, and *G. elata* growth. **(B)** HPDA25 increased fresh weight of *A. gallica* rhizomorphs. **(C)** HPDA25 increased fresh weight of *G. elata*. Control, *G. elata* co-cultured with *A. gallica*. Data are shown as means ± SE. *P < 0.05; **P < 0.01.

Furthermore, co-culturing HPDA25 with the symbiotic *G. elata*–*A. gallica* system (Figure 4A) also increased the fresh weight of *A. gallica* rhizomorphs (2.15-fold) and *G. elata* tubers (19.00-fold) (Figures 4B,C). These results demonstrate that HPDA25 could promote the growth of both *A. gallica* and *G. elata*.

### HPDA25 Produced Indole-3-Acetic Acid and Promoted the Growth of *A. gallica*

**FIGURE 5:**
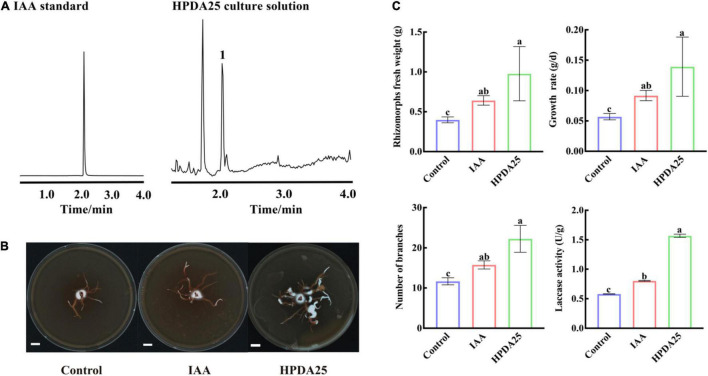
HPDA25 secreted 2.6 indole-3-acetic acid (IAA) and promoted *A. gallica* growth. **(A)** IAA was detected by ultra-performance liquid chromatography coupled to a triple quadrupole mass spectrometry. **(B)** *A. gallica* cultured in different mediums. **(C)** IAA increased rhizomorph’s fresh weight, growth rate, branching, and extracellular laccase activity of *A. gallica*. Control, *A. gallica* cultured alone in medium. Data are shown as means ± SE. Different letters indicate a significant difference at *P* < 0.05 level.

Auxin treatment stimulates AM fungal growth, mycorrhization formation (Gutjahr, 2014), and arbuscule formation (Guillotin et al., 2017). It has also been reported that *Rahnella* spp. produced IAA and interacted with AMF extraradical mycelium (Zhang et al., 2019; Yuan et al., 2020). To further investigate the mechanism of HPDA25 promoting the growth of *A. gallica* and *G. elata*, IAA content was measured in HPDA25 fermentation solution using ultra-performance liquid chromatography coupled to a triple quadrupole mass spectrometry, and the standard curve data for IAA are shown in Supplementary Table 6. A peak attributed to IAA was detected in the fermentation solution of the HPDA25 (Figure 5A), with 16.24 ng produced in 1 ml of fermentation solution (Figure 5B).

To further confirm the effect of IAA on the growth of *A. gallica*, an equal amount (approximately 16.24 ng/ml) of exogenous IAA was added to the medium to replace HPDA25. The results clearly showed that the supplement of exogenous IAA led to a 1.61-fold increase of the fresh weight of rhizomorphs, 1.61-fold growth rate, 1.35-fold rhizomorph branching, and 1.38-fold extracellular laccase activity of *A. gallica* (Figure 5C). Both exogenous IAA and co-culturing HPDA25 treatment substantially improve the growth of *A. gallica*, revealing that IAA produced in HPDA25 may be the major contributor promoting HPDA25-mediated *G. elata* growth.

### Differentially Expressed Genes in HPDA25-Mediated Promotion of *A. gallica* Growth

**FIGURE 6:**
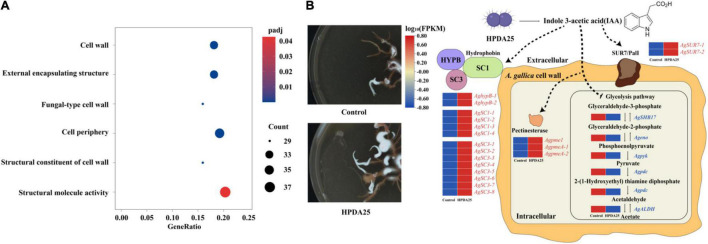
Mechanism of HPDA25 promotes growth of *A. gallica*. **(A)** Number of DEGs in *A. gallica* co-cultured with HPDA25 in GO annotation. **(B)** Mechanism of HPDA25-mediated promotion of *A. gallica* growth. Upregulated DEGs were in red words; downregulated DEGs were in blue words.

To explain the growth-promotion mechanism of HPDA25, we compared gene-expressed levels between *A. gallica* cultured alone and that co-cultured with HPDA25. A total of 4,658 upregulated DEGs and 2,669 downregulated DEGs of *A. gallica* co-cultured with HPDA25 were identified (Supplementary Figure 3). The results of GO annotation showed that upregulated DEGs are related to the structural constituent of the cell wall and structural molecule activity in molecular function (Figure 6A and Supplementary Table 7), and downregulated DEGs are related to glycolysis/gluconeogenesis (Supplementary Table 8). Twenty-four DEGs that encode hydrophobin (HFBs), Sur7/PalI family transmembrane protein, pectin methylesterase (PME), enolase (ENO), pyruvate kinase (PYK), aldehyde dehydrogenase (ALDH), or pyruvate decarboxylase (PDC) were selected to perform qRT-PCR (Supplementary Table 9), and the qRT-PCR results were consistent with the results of transcriptome analysis (Supplementary Figure 4).

## Discussion

Plants directly face various environmental challenges and thus have to recruit beneficial bacteria to cope with biotic and abiotic stress (Li et al., 2021). Mycorrhizal microbiomes are essential for exploring interactions between mycorrhizal fungi and their surrounding environments (Bahram et al., 2020). In this study, we investigated the mycorrhizosphere bacterial diversity and identified three *Rahnella* spp. strains (HPDA25, SBD3, and SBD11), which are beneficial for the increase of the yield and tuber size of *G. elata* (Supplementary Figure 2). *Rahnella aquatilis* is widely distributed (Yuan et al., 2020), and plants can recruit *Rahnella* as beneficial bacteria (Li et al., 2014). We also found that cultivating *G. elata* enhances the relative abundance of *Rahnella* but reduces bacterial diversity in cultivated soil samples, indicating that *G. elata* could only recruit beneficial *Rahnella* for its growth.

*Rahnella aquatilis* adapts to a variety of environments, which may be related to the ability of resistance (Yuan et al., 2020). To avoid damaging ecosystems, *G. elata* cultivation is shifting from woodland to farmland, and we also found that the increased relative abundance of *Rahnella* was higher in soil samples of farmland cultivating *G. elata* than those of woodland (Supplementary Table 4), suggesting that *G. elata* could recruit more beneficial *Rahnella* to accelerate the establishment of a symbiotic relationship between *A. gallica* and *G. elata* in an adverse environment.

As the third component of plant–mycorrhizal fungal–bacterial symbiosis, bacteria that are associated with mycorrhizal fungi (hypersphere) also play a critical role in mycorrhizal function (Agnolucci et al., 2020). In recent years, an intimate cooperative relationship between AM fungi mycelium and bacteria has been observed *via* microscopic observation (Scheublin et al., 2010) and molecular analyses (Wang et al., 2016). These bacterial communities could affect the growth of mycelia and the formation of mycorrhiza (Kim et al., 2013), promote the colonization of AMF (Omirou et al., 2016), and play a role in the material exchange between mycelia and plant host (Emmanuel and Babalola, 2020). However, there are few reports on the function of beneficial bacteria in OMF. *G. elata* is a completely heterotrophic plant and does not perform photosynthesis, and its growth entirely depends on *A. gallica*, an OM fungus. The pathogenicity of *A. gallica* is associated with plant cell wall degrading enzymes and invasive mycelium or rhizomorphs (Sahu et al., 2021). This indicates that *A. gallica* enter directly into the epidermal cell walls with short invasive mycelium to exchange nutrients from *G. elata* (Sella et al., 2015). Our results showed that *Rahnella* HPDA25 isolated from mycorrhizosphere soil samples of *G. elata* enhanced rhizomorph growth and the extracellular laccase activity of *A. gallica*, which could help *A. gallica* to infect wood or *G. elata* and promote the growth of *G. elata*.

The results of RNA-seq and qRT-PCR showed that *Rahnella* HPDA25 promoted the expression level of hydrophobin genes. Among HFBs, hydrophobin SC3 mediated the formation of aerial mycelium (Van Wetter et al., 1996) and aided the maturation of the fungal cell wall (Whiteford and Spanu, 2002). Therefore, *Rahnella* HPDA25 may promote cell wall maturation and rhizomorph growth of *A. gallica* by increasing the gene expression level of hydrophobin SC3 protein. Hydrophobin HYPB was first identified in *Agaricus bisporus* (Van Wetter et al., 1996), which enhanced the adherence of fungal structures to the surface of a host organism, thereby facilitating pathogenesis (Piscitelli et al., 2017). Hydrophobins HYPB may be involved in the symbiosis process, and the expression level of the hydrophobin gene was increased when mycorrhizal symbiosis was established in *Tricholoma terreum*–*Pinus sylvestris* symbiosis and *Pisolithus tinctorius–Eucalyptus globulus* symbiosis (Mankel et al., 2000, 2002). Fungal pectinases are also important during the infection of plants (Sella et al., 2015). Hence, the stimulated expression of hydrophobin HYPB and PME by *Rahnella* HPDA25 may help establish the symbiosis of *A. gallica* and *G. elata.* The downregulated DEGs, including ENO, PYK, ALDH, and PDC, were the key enzymes in the glycolysis pathway (Supplementary Table 8). A recent study reported that accelerated glycolysis might inhibit fungi mycelial growth (Yan et al., 2020). Thus, *Rahnella* HPDA25 may also promote *A. gallica* rhizomorph growth by inhibiting the glycolysis of *A. gallica. Rahnella* HPDA25 has been shown to stimulate mycelial development and fungal pathogenicity, suggesting that it may aid *G. elata* and *A. gallica* in improving nutrient absorption and increasing both yield and tuber size *in G. elata.*

The bacteria may promote the growth of plants and fungi through secreting IAA (Defez et al., 2019). Low concentrations of exogenous IAA modulate arbuscule formation in AM symbiosis (Chen et al., 2021). In our study, 16.24 ng/ml IAA was detected in the fermentation solution of *R. aquatilis* HPDA25 isolated from mycorrhizosphere soil samples of *G. elata*. Co-cultured *A. gallica* with HPDA25 or 16.24 ng/ml exogenous IAA increased the rhizomorph branching and growth rate of *A. gallica*, and HPDA25 co-cultured with *A. gallica* significantly increased the IAA content of *A. gallica* (Supplementary Figure 5). Thus, we speculate that the mechanisms of HPDA25 improving the growth of A. gallica and the nutrient uptake of G. elata are underlying the IAA regulation (Figure 6B).

## Conclusion

Orchid mycorrhizal fungi form strict symbiotic relationships with most orchid plants and play critical roles in plant growth and nutrition. In this study, we quantified the diversity of OM bacteria in the symbiosis system of *G. elata* and *Armillaria* and found that co-inoculation of both mycorrhizal fungi and PGPB was beneficial to the growth of *A. gallica* and *G. elata*. *G. elata* could regulate the mycorrhizosphere bacterial communities, especially by recruiting *Rahnella* HPDA25. The IAA-secreted HPDA25 promoted the growth of *A. gallica*, regulated the nutrient uptake of *A. gallica* and *G. elata*, and enhanced the yield and tuber size of *G. elata*. These results provide new insights into OM symbiosis and *the* cultivation of *G. elata*.

## Data Availability Statement

The datasets presented in this study can be found in online repositories. The names of the repository/repositories and accession number(s) can be found in the article/Supplementary Material.

## Author Contributions

YY and LH designed the experiments. TL conducted the experiments. YZ and JZ prepared samples for amplicon sequencing. ZH performed the bioinformatics. TL and PH contributed to data analysis. TL and YY wrote the manuscript. YJ and XL performed the identification of the plant resource. All authors read and approved the manuscript.

## Conflict of Interest

The authors declare that the research was conducted in the absence of any commercial or financial relationships that could be construed as a potential conflict of interest.

## Publisher’s Note

All claims expressed in this article are solely those of the authors and do not necessarily represent those of their affiliated organizations, or those of the publisher, the editors and the reviewers. Any product that may be evaluated in this article, or claim that may be made by its manufacturer, is not guaranteed or endorsed by the publisher.

## Footnotes

1
http://cutadapt.readthedocs.io/en/stable/

2
http://www.drive5.com/uparse/

3
http://www.arb-silva.de/

4
http://www.drive5.com/muscle/

5
https://magic.novogene.com

6
https://www.ezbiocloud.net/

7
https://blast.ncbi.nlm.nih.gov

8
https://ngdc.cncb.ac.cn/

9
http://www.primer3plus.com/cgi-bin/dev/primer3plus.cgi

10
https://hiplot.com.cn

11
https://ngdc.cncb.ac.cn/gsa/browse/CRA004378

12
https://ngdc.cncb.ac.cn/gsa/browse/CRA004382
