# Supplementary material for: Mycorrhizosphere Bacteria, Rahnella sp. HPDA25, Promotes the Growth of Armillaria gallica and Its Parasitic Host Gastrodia elata
Source: Front Microbiol. 2022 Mar 17;13:842893. doi: 10.3389/fmicb.2022.842893 (PMC8993504; doi:10.3389/fmicb.2022.842893)
Supplement: Supplementary file 1 [file Data_Sheet_1.pdf]

Supplementary Material

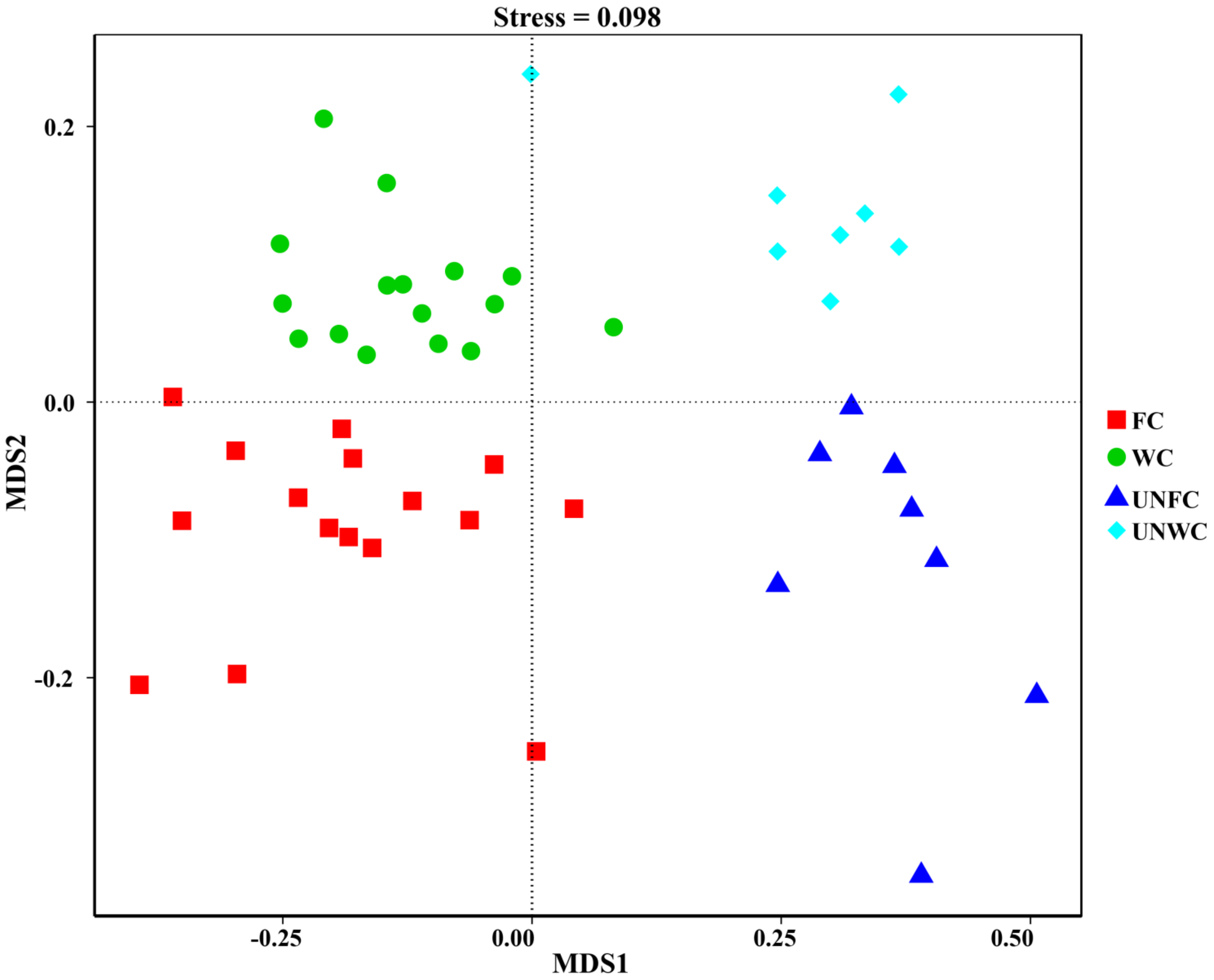

Supplementary Figure 1. Non-metric multidimensional scaling (NMDS) analysis of the soil samples. WC, mycorrhizosphere from woodland soil cultivating *G. elata*; FC, mycorrhizosphere from farmland soil cultivating *G. elata*; UNWC, mycorrhizosphere from woodland soil uncultivating *G. elata*; UNFC, mycorrhizosphere from farmland soil uncultivating *G. elata*.

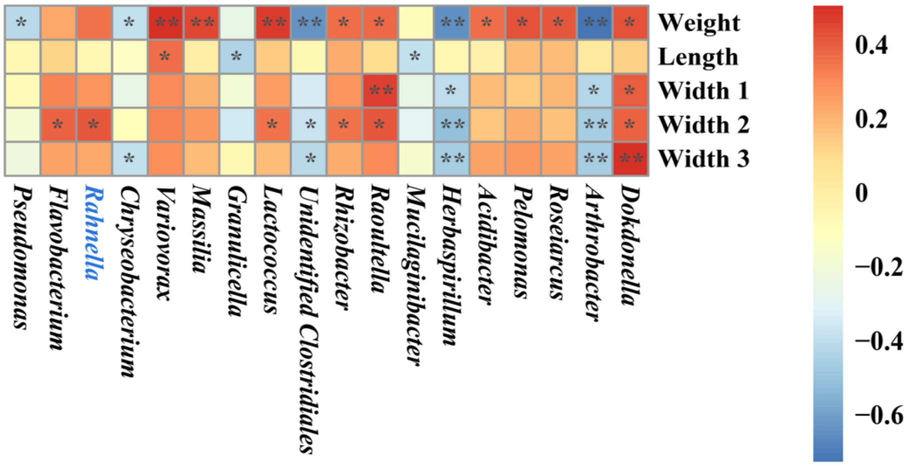

Supplementary Figure 2. Spearman correlation analysis of mycorrhizosphere bacteria and *G. elata* tuber agronomic traits. In the heat map, the value of the Spearman correlation coefficient (r) is between -1 and 1. r < 0, negative correlation; r > 0, positive correlation. *, $P < 0.05$; **, $P < 0.01$.

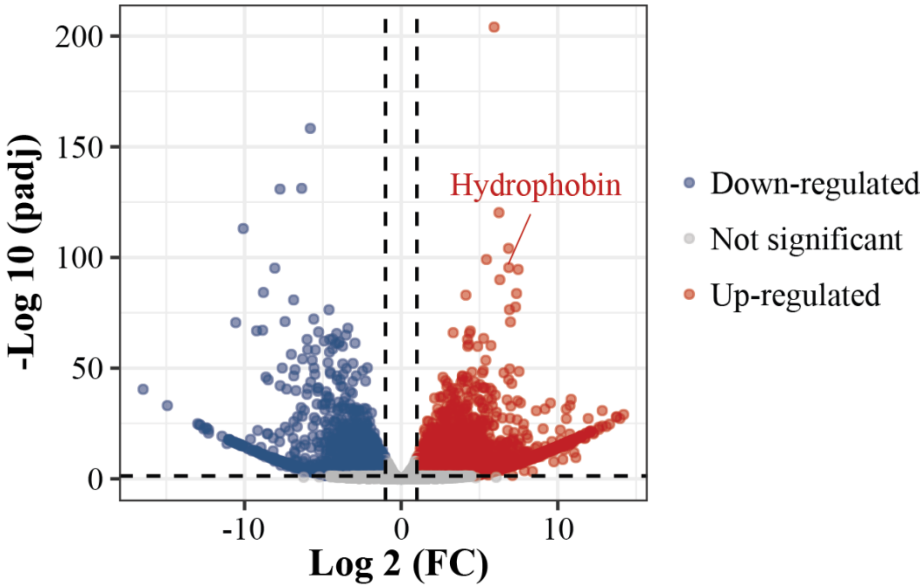

Supplementary Figure 3. DEGs in *A. gallica* co-cultured with HPDA25 and *A. gallica* cultured alone. Adjusted *P*-value (padj) ≤ 0.05, fold change (log2FC) ≥ 1.

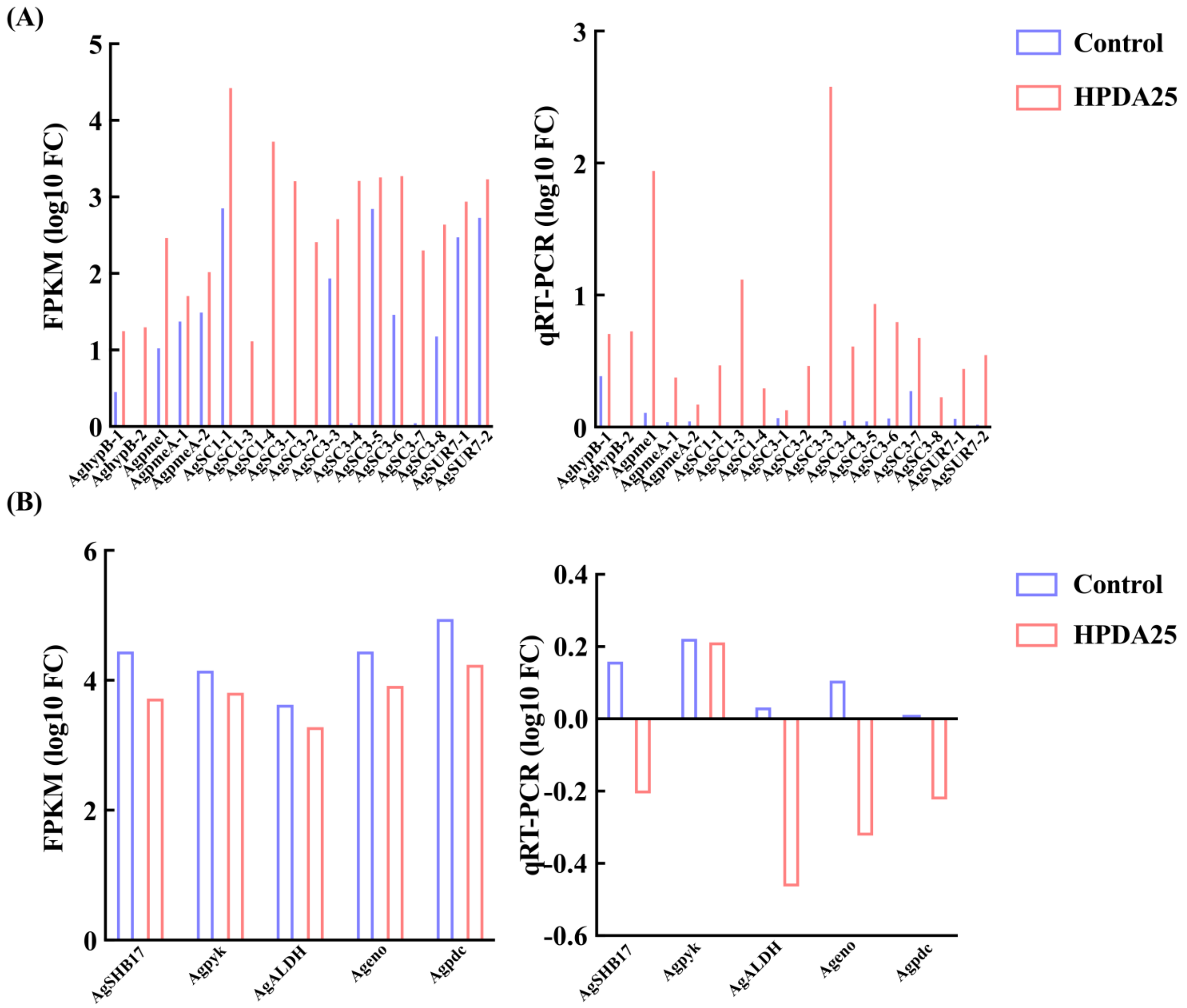

Supplementary Figure 4. Expression level of the DEGs in *A. gallica* co-cultured with HPDA25. (A), Up-regulated DEGs. (B), Down-regulated DEGs. Control, *A. gallica* cultured in PDA medium. HPDA25, *A. gallica* co-cultured with HPDA25.

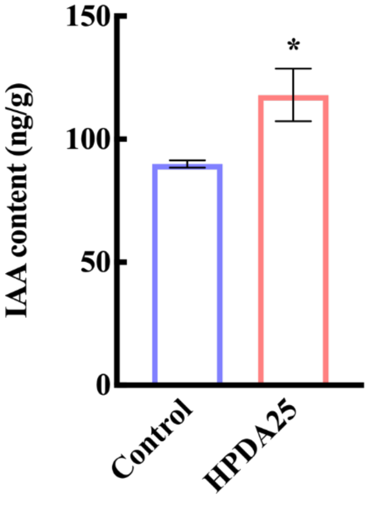

Supplementary Figure 5. IAA contents in *A. gallica* co-cultured with HPDA25 and in *A. gallica* cultured alone. Control, *A. gallica* cultured alone in the medium. *, $P < 0.05$. Data are shown as means ± SE.

Supplementary Table 1. Quantitative PCR primers.

Gene name	Primers (5'-3')	
AghypB-1	ATTACTGCCGGAGTTGGTCTTA	CTCCAACATTGATGGGAGTACAC
AghypB-2	CTCGCATCTCGACTTTTACTCTC	GGACTCTGAGTAGAAGTACAGCA
Agpme1	GACTACATCTTTGGCCAATACGC	ACTTACTCCACTCGATGATGAGC
AgpmeA-1	CCTGTCAGAGGCACTAGATGATA	ACTATCGTCAGTCGTAGTCTCAC
AgpmeA-2	AGCTAGAGTAGGGAAAAGGTCTG	GTCCCAGAGTCTTGTGTAGTACA
AgSC1-1	TTGCCAGCCTTTTGGGACT	TGTTCTCGCAGCAGACGGT
AgSC1-2	CGCCGTTGATTCTCTATCCTAGT	GTCTGTCAAAGCATAGAACGACG
AgSC1-3	CAGTGTTGCGATTCTACTCAGAG	AAACACCAACATTGCCAGTGAG
AgSC1-4	GGGCGTTGAATCATTACCTATCG	CAAGAGAGCAACGGAAGAAAGAC
AgSC3-1	ATCTCACTGCTAATGTCGGTGT	CGAGAGCAATAAGACCGTTGAAG
AgSC3-2	TTCCCCCGTTAAGACTCTCTTAG	CACCAATACCGATGACACTGATC

AgSC3-3	GGAGATTTTC GTGATTCGG AGTG	CATTGAGTCCGTG TCTGAGAAAG
AgSC3-4	ATGTTTGCTC GTATCTCCTT CA	CATTGGACAGTAC CAGTCGC
AgSC3-5	GGTCAACTC ACTGGAAAT ATCGG	CCAAGAGAAATAA GGCCGTTGA
AgSC3-6	TCGGTCAACT CACTGGAAA T	GTTACAGCAGACG ACTTGGTTA
AgSC3-7	CAGTGTTGTG CATCTACTCA GAG	CATTACCAGTGAG TTGACCAACG
AgSC3-8	CTAACTGTAT ACTGGGCTC GACC	CTACCGGACAGTA AGAGCGTAAA
AgSUR7-1	CTGGGTTACA CTTTCGATAG CAC	CAGGGAAGATAG GAAGGTCAAGA
AgSUR7-2	CGTGGAACT CTGTGTATTT CCTC	CCAGCAGTGTTAA AGTCGTATCC
AgSHB17	AGAAGGACT TGACAGACG AGTTC	GTCAGGCAATCCA AATACGGAAG
Agpyk	GTCAACACT GTCGAGAAA CTTGG	GTGTTGTCAATGA CACTCTGGTG
AgALDH	GCACGAAAG GCCTTCAATA CTAC	GTTCTTGCGCATC TCTCTCAATC

Ageno	TACTCTTCCC GTCGTCAATT CTC	CTTCAGGATTCGC TGTCGTTTC
Agpdc	CACTCAAAT ACGATGTTCC GCTC	CAGTTTCTGCGAC GATAACATCC
18S	ACCACATCC AAGGAAGGC AGCA	CCAAGAGGTAAGA CCCAGTCAGACAG TA

Supplementary Table 2. Diversity indices of soil bacterial communities (mean ± SE).

Soil	Good's coverage	Shannon	Simpson
WC	0.992 ± 0.001	7.648 ± 0.731	0.975 ± 0.018
FC	0.993 ± 0.001	6.923 ± 1.095	0.939 ± 0.061
UNWC	0.992 ± 0.000	9.311 ± 0.574**	0.994 ± 0.005*
UNFC	0.993 ± 0.001	9.076 ± 0.504$^{\#\#}$	0.994 ± 0.004$^{\#}$

WC, mycorrhizosphere from woodland soil cultivating *G. elata*; FC, mycorrhizosphere from farmland soil cultivating *G. elata*; UNWC, mycorrhizosphere from woodland soil uncultivating *G. elata*; UNFC, mycorrhizosphere from farmland soil uncultivating *G. elata*. *, compared with WC; $^{\#}$, compared with FC; *, $P < 0.05$; **, $P < 0.01$; $^{\#}$, $P < 0.05$; $^{\#\#}$, $P < 0.01$.

Supplementary Table 3. Analysis of similarities (ANOSIM) of soil bacteria.

Group	*R*-value	*P*-value
WC-UNWC	0.9217	0.001^{**}
FC-UNFC	0.9343	0.001^{**}
WC/FC-UNWC/UNFC	0.889	0.001^{**}

WC, mycorrhizosphere from woodland soil cultivating *G. elata*; FC, mycorrhizosphere from farmland soil cultivating *G. elata*; UNWC, mycorrhizosphere from woodland soil uncultivating *G. elata*; UNFC, mycorrhizosphere from farmland soil uncultivating *G. elata*. **, $P < 0.01$.

Supplementary Table 4. Genera with increased relative abundance in *G. elata*-cultivated soil samples.

Genera	WC	UNWC	FC	UNFC
Pseudomonas	11.27%	1.36%	22.05%	0.75%
Novosphingobium	5.75%	1.12%	6.35%	0.13%
Flavobacterium	5.96%	2.22%	4.11%	3.00%
Rahnella	1.39%	0.94%	0.06%	0.01%
Variovorax	4.08%	1.18%	2.48%	0.73%
Bradyrhizobium	2.77%	2.05%	2.83%	2.34%
Massilia	2.13%	0.46%	1.14%	0.21%
Collimonas	2.09%	0.22%	1.81%	0.08%
Duganella	1.70%	0.27%	1.63%	0.13%
Raoultella	0.51%	0.41%	0.32%	0.01%
Limnohabitans	0.76%	0.10%	0.22%	0.06%
Dyella	0.88%	0.42%	1.13%	0.11%
Rhizobacter	1.08%	0.40%	0.81%	0.21%
Mucilaginibacter	0.40%	0.05%	0.47%	0.03%

WC, mycorrhizosphere from woodland soil cultivating *G. elata*; FC, mycorrhizosphere from farmland soil cultivating *G. elata*; UNWC, mycorrhizosphere from woodland soil uncultivating *G. elata*; UNFC, mycorrhizosphere from farmland soil uncultivating *G. elata*.

Supplementary Table 5. Identification of *Rahnella* isolates using EzBioCloud.

No.	Stain	Accession	Pairwise similarity (%)
HPDA25	*Rahnella aquatilis* CIP 78.65	CP003244	99.77
SBD3	*Rahnella aceris* SAP-19	MN737201	99.86
SBD11	*Rahnella aceris* SAP-19	MN737201	100.00

Supplementary Table 6. Standard curve data for indole-3-acetic acid (IAA) content.

Ingredient	Linear relationship	R^2	Linear range ng/mL
IAA	$Y = 17.24546\ X\text{-}32475.2$	0.99909	0.05×10^{-3}-1000

Supplementary Table 7. GO annotations of up-regulated DEGs in *A. gallica*.

Category	GOID	Annotations	Padj	Gene number
CC	GO:0009277	Fungal-type cell wall	0.000	29
CC	GO:0005618	Cell wall	0.000	33
CC	GO:0030312	External encapsulating structure	0.000	33
CC	GO:0071944	Cell periphery	0.000	35
MF	GO:0005199	Structural constituent of cell wall	0.000	29
MF	GO:0005198	Structural molecule activity	0.043	37

CC: Cellular component, MF: molecular function.

Supplementary Table 8. KEGG annotations of down-regulated DEGs in *A. gallica.*

KEGGID	Annotations	Padj	Gene number
lbc00010	Glycolysis / Gluconeogenesis	0.000	10

Supplementary Table 9. Expression level of DEGs in *A. gallica* co-cultured with HPDA25 (mean ± SE).

Gene name	Locus name	Fpkm		qPCR	
		Control	HPDA25	Control	HPDA25
AghypB-1	Ag12055	0.445 ± 0.349	2.734 ± 1.570	2.436 ± 1.835	5.077 ± 3.444
AghypB-2	Ag22253	-	3.072 ± 1.819	1.074 ± 0.392	5.316 ± 3.060
Agpme1	Ag23760	0.300 ± 0.179	8.162 ± 4.422	1.284 ± 0.805	87.507 ± 13.093
AgpmeA-1	Ag08229	0.900 ± 0.197	1.902 ± 0.659	1.096 ± 0.476	2.373 ± 1.008
AgpmeA-2	Ag19118	0.918 ± 0.159	3.065 ± 1.811	1.108 ± 0.525	1.486 ± 0.534
AgSC1-1	Ag04607	110.258 ± 77.062	4038.754 ± 2544.147	0.860 ± 0.819	2.947 ± 1.786
AgSC1-2	Ag12888	0.051 ± 0.073	28.567 ± 4.882	0.850 ± 0.313	0.956 ± 0.290
AgSC1-3	Ag13060	-	1.299 ± 1.081	1.260 ± 0.369	13.170 ± 0.456
AgSC1-4	Ag23757	0.052 ± 0.074	360.411 ± 96.377	0.938 ± 0.532	1.962 ± 0.855

AgSC3-1	Ag12889	0.159 ± 0.225	246.180 ± 25.733	1.174 ± 0.616	1.348 ± 0.319
AgSC3-2	Ag19138	-	13.566 ± 4.808	1.248 ± 0.834	2.912 ± 1.105
AgSC3-3	Ag21588	8.683 ± 1.672	50.827 ± 27.514	1.028 ± 0.250	378.429 ± 153.672
AgSC3-4	Ag23520	0.176 ± 0.149	254.636 ± 101.618	1.120 ± 0.504	4.095 ± 1.476
AgSC3-5	Ag23517	106.702 ± 15.135	272.381 ± 118.129	1.109 ± 0.522	8.595 ± 0.802
AgSC3-6	Ag23620	4.427 ± 1.269	281.308 ± 120.306	1.162 ± 0.591	6.264 ± 3.115
AgSC3-7	Ag23776	0.052 ± 0.042	9.297 ± 2.664	1.876 ± 0.022	4.734 ± 1.715
AgSC3-8	Ag24594	0.270 ± 0.062	7.737 ± 3.110	1.017 ± 0.186	1.684 ± 0.530
AgSUR7-1	Ag03868	23.478 ± 2.767	67.262 ± 41.846	1.160 ± 0.613	2.762 ± 1.039
AgSUR7-2	Ag15686	36.475 ± 4.273	114.026 ± 21.459	1.045 ± 0.320	3.521 ± 1.364
AgSHB17	Ag15981	1009.220 ± 374.569	185.746 ± 30.158	1.452 ± 0.312	0.617 ± 0.228
Agpyk	Ag01118	348.850 ± 14.275	157.840 ± 19.497	1.680 ± 0.624	1.641 ± 0.437

AgALDH	Ag02259	102.206 ± 15.391	45.687 ± 1.380	1.085 ± 0.460	0.341 ± 0.153
Ageno	Ag00110	1174.885 ± 368.872	343.801 ± 63.409	1.287 ± 0.947	0.472 ± 0.139
Agpdc	Ag03338	2709.616 ± 983.321	522.409 ± 224.945	1.035 ± 0.278	0.594 ± 0.159

Control, *A. gallica* cultured in PDA medium. HPDA25, *A. gallica* co-cultured with HPDA25.
